# Open Abdominal Aortic Repair to Treat Perigraft Seroma after Endovascular Aortic Repair with Endologix AFX2 Endograft

**DOI:** 10.3400/avd.cr.21-00104

**Published:** 2021-12-25

**Authors:** Masamichi Ozawa, Masaki Hamamoto, Taira Kobayashi

**Affiliations:** 1 Department of Cardiovascular Surgery, JA Hiroshima General Hospital, Hatsukaichi, Hiroshima, Japan

**Keywords:** abdominal aortic aneurysm, endovascular aortic repair, perigraft seroma

## Abstract

A 75-year-old man with an abdominal aortic aneurysm underwent endovascular aortic repair (EVAR) using an AFX2 endograft with no endoleaks. Nevertheless, the aneurysmal sac increased by 8 mm at 24 months after EVAR despite no detectable endoleaks. Open surgical treatment was performed because of the risk of rupture. Intraoperative findings of much viscous cloudy fluid with no blood flow in the sac suggested that perigraft seroma resulted in sac enlargement. The endografts were replaced by a Dacron graft. Perigraft seroma should be considered as a cause of sac growth after EVAR with AFX2 when there are no detectable endoleaks.

## Introduction

Perigraft seroma (PGS) complicates open abdominal aortic aneurysm (AAA) repair by using a vascular prosthesis with a relatively low incidence.^1,2)^ To date, several reports have elucidated the possible etiology and pathogenesis of PGS after open AAA repair. Conversely, there have been few reports regarding PGS associated with endovascular aortic repair (EVAR).

Here, we report our experience of PGS after EVAR using an Endologix AFX2 endograft, which comprises extended polytetrafluoroethylene (ePTFE) graft.

## Case Report

**Figure 1:**
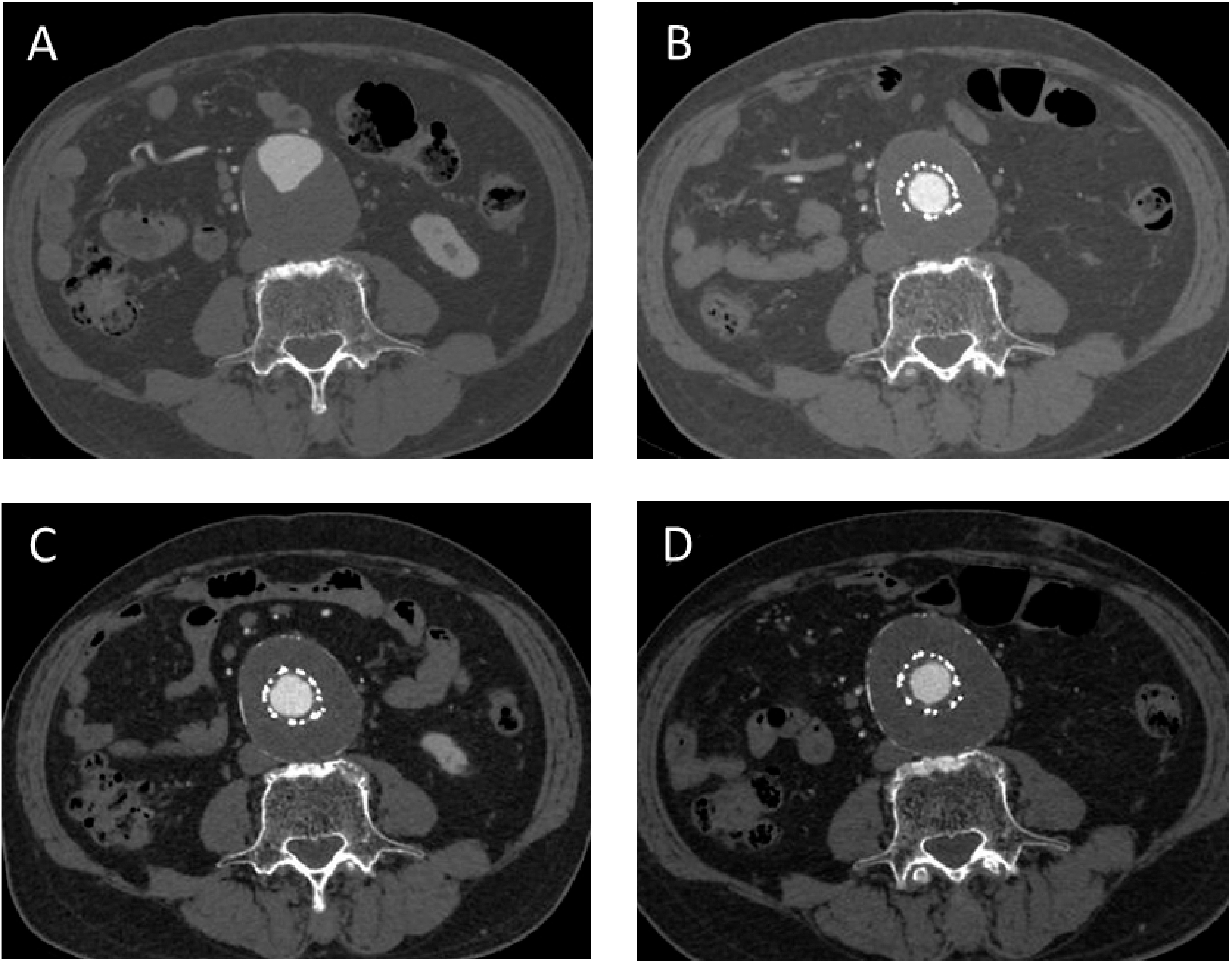
Fig. 1 Contrast-enhanced computed tomographic scan showing (**A**) a maximal aneurysm diameter of 65 mm before endovascular aortic repair (EVAR), (**B**) a maximal diameter of 65 mm with no signs of endoleak at 6 months after EVAR, (**C**) an unchanged maximal diameter of 66 mm with no signs of endoleak at 12 months after EVAR, and (**D**) an increased maximal diameter of 73 mm with no signs of endoleak at 24 months after EVAR.

A 75-year-old man with a diagnosis of 65 mm AAA (**Fig. 1A**) underwent EVAR with an AFX2 endograft (Endologix Inc., Irvine, CA, USA) of the main body (BEA25-100/I20-40) and a suprarenal proximal extension (A28-28/C95-O20) in 2018. The patient had suitable anatomy for EVAR within the instructions for use. The procedure was successful with no endoleaks on completion angiography. Contrast-enhanced computed tomography (CT) showed no signs of endoleaks with a maximal aneurysm diameter of 65 mm at 6 months and 66 mm at 12 months after EVAR (**Figs. 1B** and **1C**). On the 24 month contrast-enhanced CT, however, the maximal diameter increased to 73 mm with no detectable endoleaks (**Fig. 1D**). Although the patient was asymptomatic with a negative result of blood test (white blood count of 7900/µL and C-reactive protein level of 0.6 mg/dL), open surgical treatment was planned because of the increasing aneurysm diameter with the potential risk of rupture.

**Figure 2:**
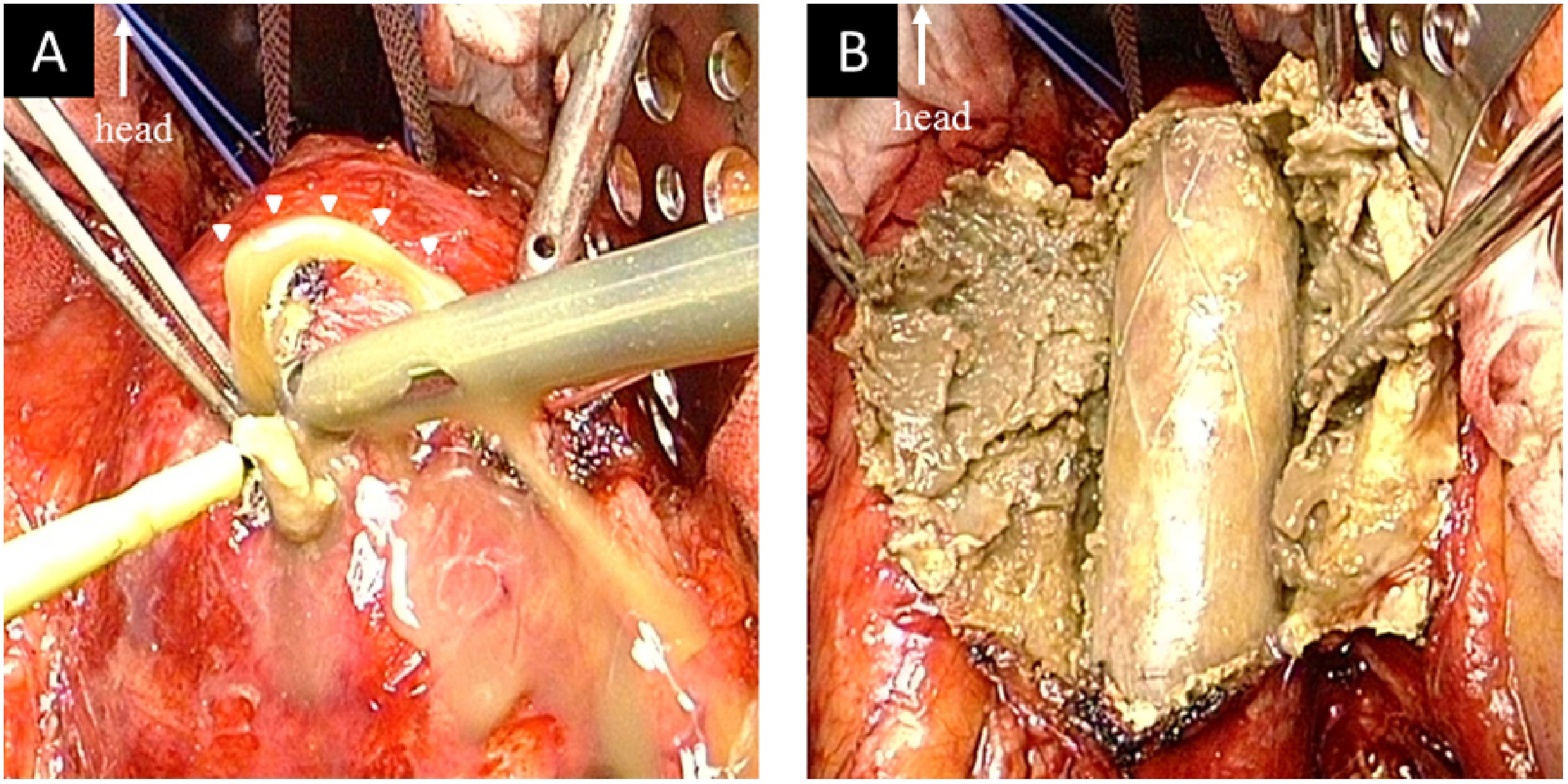
Fig. 2 Intraoperative photos showing (**A**) viscous cloudy fluid spouted out from the aneurysmal sac just after the incision (white arrowheads) and (**B**) straw-colored viscous material covering the endograft and neither blood flow into the sac nor blood staining of the endograft.

We performed median laparotomy and found that the aneurysm was pulsative without any sac appearance of inflammation and infection. The suprarenal aorta and the bilateral external and internal iliac arteries were carefully dissected and encircled by the Teflon tape. After systemic heparinization, the pressurized aneurysm sac was incised without an aortic clamp, and a large amount of viscous cloudy fluid spouted from the sac (**Fig. 2A**). The endografts were surrounded by straw-colored viscous material. Blood flow into the sac was not recognized (**Fig. 2B**). The suprarenal abdominal aorta and the bilateral internal and external iliac arteries were clamped for endograft explant. The suprarenal aortic clamp did not interfere with the suprarenal proximal extension. The main body and the suprarenal proximal extension were easily removed. The aortic clamp was moved to the infrarenal position. Renal ischemic time was 60 s. Conventional graft replacement by using a bifurcated Dacron graft was performed. The remaining sac was tightly closed around the graft.

The bacterial culture of the sac contents was negative. The postoperative course was uneventful, and the patient was discharged home on postoperative day 16. PGS has not recurred during 12 months after graft replacement.

## Discussion

Aneurysm sac enlargement after EVAR is a crucial finding because it means treatment failure and can cause sac rupture.^3)^ Sac enlargement is associated with any types of endoleaks and endotension (endoleak type V). Endoleaks can be diagnosed on a contrast-enhanced CT scan. By contrast, endotension can be diagnosed by no evidence of endoleaks despite increased aneurysmal sac. Endotension is associated both with transudate accumulation within the sac due to an endograft porosity and with pressure transmission through an endograft and intrasac thrombus. The former case is called “perigraft hygroma”^4)^ or “PGS.”^5)^ In our case, sac enlargement with a diameter increase of 8 mm per year was confirmed on a routine CT scan without any types of endoleaks after successful EVAR with AFX2. CT also showed homogeneous density in the sac, which seemed to be different from the findings of occult endoleaks. Much viscous cloudy fluid without no blood flow in the aneurysmal sac was recognized during surgery. Intrasac pressure was not measured, but fluid evacuation under tension implied increased intrasac pressure. On the basis of these clinical conditions, we eventually diagnosed PGS during the surgery.

PGS is well recognized after open AAA repair mainly using a PTFE graft.^1,2)^ The causative factors have been reported to be a low-grade infection, an immunologic reaction to the graft material, and an extravasation of plasma fluid through the graft.^6)^ PGS develops even after EVAR. Cho et al. reported late aneurysm enlargement after EVAR with an Excluder bifurcated endograft (W. L. Gore & Associates Inc., Flagstaff, AZ, USA) made of ePTFE fabric. The authors showed that nine of 45 patients developed late sac enlargement with no active endoleak.^7)^ Nano et al. reported a case of sac enlargement caused by seroma after EVAR with a PowerLink endograft (Endologix Inc., Irvine, CA, USA) made of ePTFE fabric, which was a former product of AFX2.^5)^ The difference between the PowerLink and AFX2 system is the graft processing methods. The AFX2 system characterizes tighter mechanical strength with helically wrapped ePTFE layers despite a reduced thickness of the graft wall, which is called the DURAPLY graft. The DURAPLY graft of AFX2 is a stronger and less permeable ePTFE graft than the first-generation graft of PowerLink. Although the exact pathogenesis of PGS with AFX2 remains unclear, direct pressure transmission through a strengthened graft material and serous fluid transudation through a thinner graft material can be the contributory mechanism of the PGS with AFX2 endograft. Risberg et al. have proposed a new mechanism of PGS. They described that thrombus degradation via stress-induced fibrinolysis and subsequent protein-rich perigraft fluid collection provoke the increased permeability of serum across the endograft.^4)^ Regarding the association between the materials of the stent graft and PGS, there have been no reports on PGS after the implantation of stent grafts made of woven polyester (Endurant (Medtronic Inc., Santa Rosa, CA, USA) or Zenith Flex (Cook Inc., Bloomington, IN, USA)). This suggests that woven polyester stent grafts seem to be rarely involved in PGS, unlike ePTFE stent grafts.

In our case, we planned to perform open surgery for the asymptomatic patient with sac growth reaching 73 mm. Generally, close observation is recommended for asymptomatic patients with stable PGS.^8)^ Symptomatic patients with increasing PGS need interventions. Interventions include open surgical procedures such as complete removal of an endograft followed by replacement and endovascular procedures such as relining.^9)^ We preoperatively considered that sac enlargement was not caused by PGS but by indiscernible, very low flow endoleaks. We were also concerned that sac enlargement could threaten the proximal and distal sealing and cause type I endoleak, which could result in sac rupture. Fortunately, the patient was not frail without any comorbidities that made invasive open surgery difficult. We eventually decided to perform definitive graft replacement.

PGS was diagnosed during surgery in our case. We did not change the surgical strategy of replacing the whole endografts because it has been reported to be a curative approach with a high success rate.^10)^ Graft replacement after EVAR includes demanding procedures such as suprarenal aortic clamp and dissection of the adhesion at the proximal and distal landing zone. Fortunately, these procedures were not so complicated and graft replacement was completed without any complications in our case. An alternative less invasive surgical option is a graft-preserved procedure such as a wrapping of the endografts with a Dacron graft and tight closure of the sac.^5)^ This operation is time-saving in comparison with graft replacement, but careful observation of the recurrence is required after the operation.

## Conclusion

We experienced a rare case of PGS after EVAR by using the AFX2 endograft. In the case of sac enlargement with no detectable endoleaks on a routine contrast-enhanced CT scan, PGS should be considered as a cause of sac growth.
